# Supplementary material for: Complexome profiling of the Chlamydomonas psb28 mutant reveals TEF5 as an early PSII assembly factor
Source: Plant Cell. 2025 Mar 17;37(6):koaf055. doi: 10.1093/plcell/koaf055 (PMC12164590; doi:10.1093/plcell/koaf055)
Supplement: koaf055_Supplementary_Data [file koaf055_supplementary_data.zip › psb28_interactive.zip!/plots/intensity/qualityPlots/peptides/_Cre12.g532100.t1.2.html]

### Cre12.g532100.t1.2

Trivial name:   
SeqCoverage [median]: 9.60  
Mass: 51.92 kDa  
  
 

| Sequence | MissedCleavage | Mass [Da] | wtIntensity | muIntensity | Score |
| --- | --- | --- | --- | --- | --- |
| LEDVVVDGSK | 0 | 1059.54 | 647.61 | 0.00 | 62.82 |
| RLMTAAQLR | 1 | 1058.60 | 0.00 | 544.58 | 82.75 |
| TGALVGGVLGAPR | 0 | 1166.68 | 702.82 | 6476.78 | 107.79 |
| VAGVSPPAPSLFSR | 0 | 1383.75 | 457.94 | 1282.97 | 104.79 |
|  |  |  | 1808.4 | 8304.3 |  |
